# Adaptive Intervention for School-Age, Minimally Verbal Children With Autism Spectrum Disorder in the Community: Primary Aim Results

**DOI:** 10.1016/j.jaac.2024.10.020

**Published:** 2025-01-24

**Authors:** Connie Kasari, Stephanie Shire, Wendy Shih, Ann Kaiser, Catherine Lord, Lynne Levato, Tristram Smith, Daniel Almirall

**Affiliations:** a UCLA, Los Angeles, California; b University of Oregon, Eugene, Oregon; c Vanderbilt University, Nashville, Tennessee; d University of Rochester, Rochester, New York; e University of Michigan, Ann Arbor, Michigan.

**Keywords:** minimally verbal, intervention, SMART design, JASP-EMT, DTT

## Abstract

**Objective:**

The goal of this study is to construct a 16-week, 2-stage, adaptive intervention consisting of DTT (Discrete Trials Training, largely considered usual care for children with autism), JASP-EMT (a blended, naturalistic, developmental behavioral intervention involving JASPER [Joint Attention, Symbolic Play, Engagement and Regulation] and EMT [Enhanced Milieu Teaching]), and parent training (P) for improving spontaneous communicative utterances in school-aged, minimally verbal autistic children. Intervention was delivered both at school (DTT, JASP-EMT) and at home (P). This article reports results for the study’s primary aim and a closely related secondary aim.

**Method:**

The study used a 2-stage, sequential, multiple-assignment randomized trial design. In stage 1 (weeks 1–6), 194 minimally verbal (<20 functional words), 5- to 8-year-old autistic children were randomized initially to DTT vs JASP-EMT (stage 1, weeks 0–6). Early vs slower response status was determined at the end of stage 1. In stage 2 (weeks 7–16), early responders were re-randomized to stay the course vs P, whereas slower responders were re-randomized to stay the course vs combined DTT+JASP-EMT). The primary aim was to test whether there was a difference between starting with DTT vs starting with JASP-EMT on average change in socially communicative utterances (SCU; primary outcome) from baseline to week 16. A secondary aim was to estimate which of the 8 prespecified interventions was most favorable (ie, the largest average SCU at week 16). The secondary outcomes were total number of novel words, joint engagement, play diversity, requesting, and joint attention gestures from independent blinded assessments.

**Results:**

There was no evidence to reject the null hypothesis of no difference between starting with DTT or JASP-EMT on primary outcome (*p* = .41). The most favorable of the 8 interventions was the adaptive intervention, which starts with DTT, augments with P for early responders, and augments with JASP-EMT for slower responders. For this adaptive intervention, average change on SCU from baseline to week 16 for this intervention was estimated to be 7.68 (95% CI = 2.13–13.24).

**Conclusion:**

The results showed no difference in treatment starting with JASP-EMT or DTT, and the differences among the 8 adaptive interventions of the secondary aim were modest. Based on these results, reflections on next steps are discussed.

## Plain language summary

There is limited research about the optimal strategy for interventions to improve spoken language outcomes among minimally verbal 5- to 8-year-old children with autism. The goal of this sequentially randomized trial of 194 children was to compare starting intervention with either a highly structured intervention (discrete trial training [DTT], considered usual care) or a blended, naturalistic, developmental and behavioral intervention (Joint Attention, Symbolic Play, Engagement and Regulation [JASPER] and Enhanced Milieu Teaching [EMT], JASP-EMT) and then changing the intervention depending on how the child responded to the initial intervention. Results at 16 weeks found that there was no difference in improvement in spoken language outcomes for children who began with either intervention first. However, an adaptive intervention was modestly better for having the greatest spoken language at the end of the study: begin with DTT, then add parent training in the home for early responders, and combine DTT and JASP-EMT for slow responders.

**Clinical trial registration information:**

Adaptive Interventions for Minimally Verbal Children With ASD in the Community (AIM-ASD); https://clinicaltrials.gov/study/NCT01751698.

Autism has a highly variable (ie, heterogeneous) phenotypic expression, even for core characteristics of social communication and language, and within subgroups such as children who are minimally verbal (MV; defined as fewer than 20 single functional words). Clinicians also expect there to be significant heterogeneity in response to intervention. A single-component intervention is unlikely to be effective long term for all MV children. On the other hand, an adaptive intervention^1–4^ that strategically sequences the provision of different components to meet children’s heterogeneous needs is likely to benefit a greater number of children. For autistic children who are MV, we know very little about how best to construct such interventions, as these children are often excluded from research studies. The aim of the current study−known as AIM-ASD (Adaptive Interventions for Minimally Verbal Children with ASD)−is to answer questions needed to construct effective adaptive interventions aimed at improving spoken language among 5- to 8-year-old autistic MV children.

Some autistic children reportedly learn spoken language after age 5 years, but the window may be small, and it is unclear whether social communication skills known to influence language in younger children are necessary precursors.^5,6^ In a review of studies of language acquisition in autistic individuals, Pickett *et al.*^7^ reported on 167 individuals who started speaking after age 5 years. Most individuals who acquired spoken language after 5 years did so by age 7. Children who acquired spoken language during this time were more likely to have nonverbal IQs over 50 and to have received behavioral interventions targeting sounds and words primarily used for requesting. Most children demonstrated single words, with only one-third progressing to phrase speech. Because these children were identified retrospectively and because the intervention focus was on spoken words, it is not known whether children exhibited social communicative skills known to precede spoken language (eg, joint attention gestures and joint engagement)^8^ or the extent that children benefited from augmentative and alternative communication (AAC) support (eg, tablet with speech-generating software).

One study of 5- to 8-year-old MV children^1^ sought to develop an adaptive intervention to guide the provision and timing of AAC (speech-generating device) in the context of a developmental and behavioral intervention that combined JASPER (Joint Attention, Symbolic Play, Engagement and Regulation)^9,10^ and EMT (Enhanced Milieu Teaching),^11^ hereafter referred to as JASP-EMT. At the start of intervention, children were randomized to AAC vs not; all children received JASP-EMT twice weekly. After 3 months of intervention (24 sessions) clinical response was assessed. Children with AAC who made slower progress (<25% improvement on a composite measure of spoken communication) were offered increased intervention dose (3 sessions per week). Children not initially assigned AAC who made slower progress were re-randomized to increased dose or augmentation with AAC. After 6 months, children offered the adaptive intervention that began with AAC+JASP-EMT had significantly more socially communicative utterances, on average, than any of the other adaptive interventions.^1,10^ Beginning with AAC+JASP-EMT also led to increased initiations of joint attention.^12^

The above-mentioned study led to additional questions, which motivated the intervention components to be studied in AIM-ASD, and its research design. First, children in the previous study entered with a minimum 24-month receptive language age; thus, it remained unclear how to intervene with MV children having more limited receptive language. Therefore, in AIM-ASD, we targeted a broader group of MV children with autism, with a minimum age of 18 months receptive language.

Second, the previous trial studied the AAC in the context of JASP-EMT only, leaving the question of whether incorporating a third communication teaching component could improve outcomes further. One approach that has fundamental differences from JASP-EMT is Discrete Trials Training (DTT),^13^ most often used with preschool-aged children. DTT is the closest intervention to being a community “standard of care” for children with ASD.^14^ That is, at age 5 years, most children with ASD have had some form of DTT in the home, clinic, or school-based services. Given its wide availability, an important set of questions for clinicians is the choice of DTT vs JASP-EMT as first-stage treatment for MV children, including whether there are some children who would benefit more from one than from the other.

A third set of questions concerned the provision of second-stage intervention, for both early and slower responders. Parent participation in intervention may consolidate early response by promoting generalization to everyday interactions, leading to improved use of functional language in home interactions.^15–17^ Longitudinally, parents’ participation in children’s intervention has predicted positive language outcomes.^15^ Therefore, for early responders to intervention, second-stage parent training (vs not) was hypothesized to improve communication outcomes. On the other hand, for MV children who are slower responders to first-stage DTT or JASP-EMT, a combined DTT+JASP-EMT intervention (vs staying the course) was hypothesized to improve outcomes.

## Specific Aims and Hypotheses

The goal of AIM-ASD was to construct a 16-week, 2-stage, adaptive intervention involving JASP+EMT (J), DTT (D), Parent Training (P), and Combined JASP-EMT + DTT (C). This article reports results for the study’s primary aim and a related secondary aim.

### Primary Aim.

The primary aim hypothesis test was to determine whether there is a difference between starting with JASP+EMT vs DTT on average change in socially communicative spontaneous utterances (SCU; the primary outcome) from baseline to week 16. Secondary outcomes include child-initiated joint engagement, number of novel words, joint attention, requesting, and play diversity. We hypothesized that adaptive interventions beginning with JASP-EMT would improve primary and secondary outcomes more than those beginning with DTT. The study’s sample size was selected to detect an average difference no smaller than 13 SCU by week 16.

### Secondary Aim.

A related secondary aim was to estimate which of the 8 prespecified adaptive interventions was the most favorable in terms of average SCU at week 16. We hypothesized that the adaptive intervention that (1) begins with JASP-EMT, (2) augments JASP-EMT with parent training for early responders, and (3) augments JASP-EMT with DTT for slower responders would improve child outcomes more than the other 7 adaptive interventions.

## METHOD

### Research Design

**FIGURE 1:**
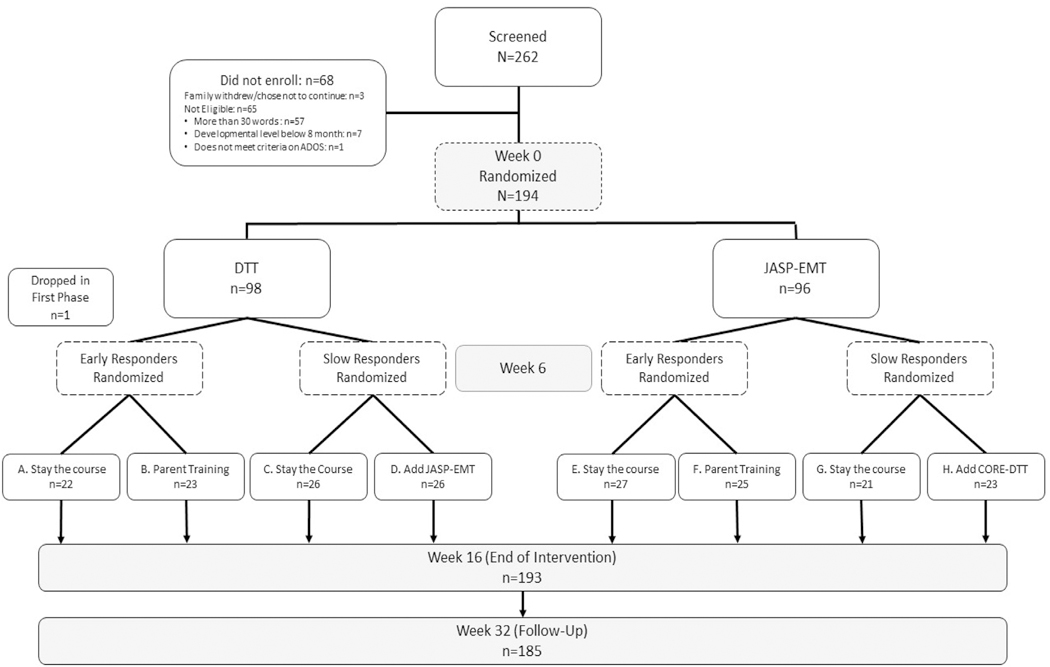
CONSORT Chart

AIM-ASD used a longitudinal, 4-site, 2-stage, sequential, multiple-assignment, randomized trial (SMART)^18,19^ design (Figure 1). All children received 16 weeks of intervention, across 2 stages; stage 1 was baseline to end of week 6, and stage 2 was the beginning of week 7 to the end of week 16. Phase durations were chosen based on previous research (6 weeks equal approximately 24 sessions [4 times per week] and sufficient for determining fast or slower response)^1^ with approximately 24 to 36 sessions sufficient for improving SCU (primary outcome).^1^

All children were randomized with equal probability at the beginning of stage 1 to JASP-EMT vs DTT (Primary Aim comparison). At the end of week 6, all children were assessed for early response vs slower response to stage 1 intervention (see Intervention Components below).

In stage 2, intervention differed depending on response status: Early responders were randomized with equal probability to continue with stage 1 intervention (ie, to stay the course) or to augment with parent training. Slower responders were randomized with equal probability to stay the course vs combined JASP-EMT+DTT.

This research design led to eight 2-stage adaptive interventions (AIs) (Table 1), which are compared as part of Secondary Aim. Individual AIs are described in the Table 1 footnotes.

The Institutional Review Board at each site approved the study protocol; the study was preregistered (NCT01751698). An external Data Safety and Monitoring Board reviewed adverse events, protocol deviations, and trial progression yearly throughout the trial.

### Randomizations

Each child was randomized twice. All randomizations occurred within site, by an independent data-coordinating center. For each child, the initial randomization occurred after the site’s project coordinator entered all necessary screening and baseline stratification data in the system (and only if the child met the study criteria). The initial randomization was stratified by the following: (1) baseline number of distinct words (NDW ≤10 vs >10); (2) Autism Diagnostic Observation Schedule (ADOS) Social Affect (≤15 vs >15); (3) child’s age (<6.5 vs >6.5 years); and (4) non-verbal IQ (<70 vs ≥70). At the end of stage 1, early responder status was entered, and the second randomization occurred automatically stratified based on the child’s stage 1 intervention.

### Participants

#### Recruitment.

Four sites (University of California at Los Angeles, Vanderbilt University, University of Rochester, and Weill–Cornell Medical Center) enrolled children. Children were recruited from the autism/developmental disorder clinics at each site, and through outreach with participating school districts.

#### Inclusion Criteria.

The study was designed to include children 4.5 to 8.5 years of age, with the following: a diagnosis of autism spectrum disorder (confirmed by research-reliable clinicians using the Autism Diagnostic Observation Schedule−2 [ADOS-2])^20^; fewer than 20 spontaneous functional words (requests and comments, both spoken and augmented) based on the baseline Natural Language Assessment (NLS), and Preschool Language Scales (PLS-5)^21^; if on medication, stable medication over the past 6 months; if a history of seizures, seizures controlled for at least 6 months; at least 1 year of previous intervention; household with at least 1 parent/caregiver willing to participate in parent training; and receptive language age equivalency of ≥18 months on the Mullen Scales of Early Learning (MSEL)^22^ Visual Reception and Fine Motor subscales, or Leiter-R.^23^

#### Exclusion Criteria.

Children were excluded if they had sensory impairments (eg, blindness, deafness) or genetic syndrome, or proficient use of an AAC modality (beyond 20 functional words).

### Intervention Components

Each of DTT, JASP-EMT, and JASP-EMT+DTT was delivered at the child’s school, 4 days per week, for approximately 45 minutes. Parent Training was conducted in the family home by the interventionist assigned to the child in stage 2. Descriptions for all intervention components are available in Supplement 1, available online.

#### DTT.

The goal of DTT is to help children learn communication skills by breaking these skills down into small steps, providing systematic direct instruction on each step, and reinforcing children (eg, with praise or access to preferred items) for demonstrating skills.^1^ DTT is often considered the closest to a “standard of practice” for the field.^14^

#### JASP+EMT.

JASP-EMT is a developmentally anchored behavioral intervention that assumes that communication develops from social interactions in which specific social engagement strategies, symbolic representations, and early communication forms are modeled and naturally reinforced by adult partner responses to the child.^1^

#### DTT+JASP-EMT.

This intervention includes strategies from each of DTT and JASP-EMT. In each 45-minute session, using a dashboard of potential intervention strategies, DTT and JASP-EMT were both applied, and individualized for the child.^24^

#### Parent Training.

Parent Training uses a combination of discussion, modeling, and live coaching support for the stage 1 intervention model (DTT or JASP-EMT). With parent training, the goal was to capitalize on early gains with school intervention to generalize intervention effects to the home, that is, beyond the school-interventionist context.

#### Early/Slower Response Monitoring.

Response monitoring is a component of each intervention (DTT and JASP-EMT) and occurs after 6 weeks of intervention. Early response vs slower response categorization was based on a 7-item Clinical Global Impressions−Improvement scale (CGI-I).^25,26^ The CGI-I was rated by the interventionist at week 6 using clinical judgment and weekly intervention data, and based on change in CG-I severity rating at baseline and week 6. “Early response” was defined as CGI-I values of 1 or 2, and “slower response” as CGI-I values of 3 to 7 at week 6. It should be noted that the CGI (often used as an outcome measure in psychiatric clinical trials was, instead, used here to monitor response, ie, was part of the intervention). A previous study has used CGI in this manner, and has found it to be valid when compared to changes in independently assessed and coded assessments.^27^

### Interventionist Training and Fidelity Monitoring

Prior to the beginning of the study, interventionists across sites were trained to fidelity (>80% on all intervention elements and quality indicators, based on a rating scale) with 2 or 3 MV children (who were not part of the randomized study). Interventionists were bachelor’s degree– level to doctoral-level therapists (education, speech, psychology) and were experienced with autistic children. Altogether, 41 different therapists participated over the trial; not all therapists delivered every intervention, nor did therapists necessarily remain the same across the child’s intervention sequence. Therapists were matched to children based on intervention fidelity and schedule. Interventionists at each site were supervised via weekly individual or group meetings (Supplement 2, available online).

### Research Measures

All primary and secondary research outcomes were collected at week 0 (baseline),week 16 (end of intervention), and week 32 (follow-up), and coded by an independent evaluator (IE) who was blinded to intervention condition and time point (Supplement 3, available online).

#### Natural Language Sample.

The Natural Language Sample (NLS)^1^ is a 21-minute standardized, video-recorded, naturalistic adult–child interaction; the child’s interventionist was not present. Independent assessors were trained on the language assessment (>0.80 reliability) with 2 or 3 children not in the study. The NLS was later transcribed using Systematic Analysis of Language Transcripts (SALT) conventions,^28^ and each verbal and nonverbal behavior was coded by an IE. IEs were trained at 1 site (Vanderbilt University) that completed transcripts across the trial. We conducted a test–retest reliability with this sample in which the assessment was conducted twice within a span of 1 week. For both SCU and number of distinct word root, the correlation between the test–retest was highly and significantly correlated (0.72 and 0.81, respectively). The average intraclass correlation coefficient (ICC) for transcript coding (SALT) was among 5 coders (number of distinct word root ICC = 0.97; SCU ICC = 0.99).

#### Primary Research Outcome.

The primary outcome was total spontaneous SCU based on the NLS, a continuous measure. SCU are unprompted, generated (nonscripted) verbal utterances that are directed to another person for the purpose of sharing information (comment) or making a request. This measure has been found to have high reliability and sensitivity to intervention in previous studies.^1^

#### Secondary Outcomes.

Five outcomes were prespecified: (1) child-initiated time jointly engaged (JE) with a caregiver, (2) number of different words (NDW), (3) number of different play types/play diversity, (4) initiating joint attention (IJA), and (5) initiating behavioral request (IBR). First is the total time in a child-initiated JE state.^1^ JE is a duration measure taken from the video-recorded Caregiver–Child Interaction (CCX). The CCX was derived from the Communication Play Protocol (CPP)^29^ including three 5-minute adapted scenes including: free play, hidden objects, and drawing. Second is the total number of novel different word roots (NDW), coded with SALT conventions from the transcribed NLS. Third, play diversity was coded from the Structured Play Assessment−Revised (SPA-R).^9,30^ Play diversity refers to the sum of the number of different types (unique actions on objects) within each play level. Finally, IJA and IBR were collected from the Early Social Communication Scales (ESCS),^31–34^ a 20-minute semistructured assessment. All assessments were conducted by blinded independent assessors.

#### Research Sample Retention Plan.

All children, once randomized were included in all analyses. Participants received compensation ($25) for attempting and/or completing assessments at baseline, week 16, and follow-up. Every effort was made to collect the primary and secondary research outcome by the IE, even if a child/family stopped the intervention. Supplement 3, available online, provides detailed descriptions of research outcomes and the study sample retention plan.

### Preplanned Analytic Plan

#### Missing Data Plan.

Missing data was multiply imputed prior to any analyses; all results presented below use standard rules for combining results across multiply imputed datasets.^35^

#### Primary Aim.

The primary aim contrasted adaptive interventions beginning with JASP-EMT vs those beginning with DTT (to evaluate the main effect of initial treatment) on change in SCU from baseline to week 16 (primary endpoint). Longitudinal mixed effects regression models were used to evaluate mean differences in the primary (SCU) and secondary (NDW, play diversity, IBR, IJA, and JE) outcomes between the 2 initial-stage interventions (JASP-EMT vs DTT). A piecewise-linear longitudinal model (with knot at week 6) was fit for each outcome. The prespecified regression included a random effect for the intercept and an unstructured within-person correlation structure for the residual errors. Each model included the following baseline covariates: site, age (months), and nonverbal IQ.

#### Secondary Aim.

For each outcome, a weighted regression analysis was used to estimate the average outcome at week 16 for each of the 8 interventions.^36,37^ By design, every child contributed data to the 8 (adaptive) interventions, depending on the interventions to which they were randomized and their early response status, requiring a weighted comparison. Empirical weights were used, which leads to estimates with greater precision; and robust standard errors took into account sampling variability in the weights^38^ (Supplement 4, available online).

To communicate the results of this analysis, the 8 interventions were ranked from the most favorable (rank = 1: maximum estimated average outcome) to the least (rank = 8). For each intervention ranked 1 to 8, the following is reported: (1) a comparison vs the average under the least favorable intervention, and (2) the standardized comparison^39^ (ie, scaled by the SD of the outcome).

### Sample Size

The planned sample size was based on a statistical power calculation for the primary aim. The primary aim hypothesis test targeted an average difference of ≥13 SCU between starting with JASP-EMT vs DTT. Differences smaller than 13 (<3 words in 5 minutes) were not considered clinically meaningful based on previous research^1^ and clinical expertise with MV autistic children. A difference >13 corresponds to detecting at least a moderate effect size (Cohen *d* = 0.5); the estimated SD of 27 was based on data from the only study of MV children that was available when AIM-ASD was designed.

To detect a difference of 13 SCU with 90% power, N = 192 was required. This calculation was based on a 2-sided, 2-sample, hypothesis test based on a type I error rate of 5%, SD = 27 on SCU, an estimated attrition rate of 10% by the primary endpoint, and a sample size of 96 randomized to each stage 1 intervention arm. Ninety percent power at N = 192 was considered conservative. First, the 10% attrition rate used to calculate the sample size was set significantly higher than in prior trials. Second, the proposed analytic plan used a repeated-measures regression, which was expected to provide increased power in proportion to the within-person correlation in SCU.^40^ Using data from the Kasari *et al.*^1^ trial, the SCU within-person correlation was estimated to be 0.60, for which power was 98%.

## RESULTS

### Intervention Retention, Study Retention, and Missing Data

Only 1 participant exited intervention early and was the only study dropout. Less than 10% of data were missing across all measures.

### Sample Descriptives

A total of 262 racially and ethnically diverse children were screened for eligibility; of these, 68 children were not eligible, resulting in a total of 194 children randomized to the initial stage 1 intervention (JASP-EMT: n = 96 vs DTT: n = 98) (Figure 1). The majority of children enrolled were male (n = 154; 79.38%) and on average 6 years of age (SD = 1.26). Table 2 shows the baseline characteristics, by intervention, for the 194 children. The average SCU at baseline was estimated as 11.17 (95% CI = 8.79–13.56). For 47 children (24%), a Leiter-R basal at 24 months could not be established; therefore, per the study protocol, the MSEL was administered.

### Interventionist Fidelity

Supplement 5 and Table S1, available online, show high fidelity scores, on average, across interventionists and intervention components, over the course of 16 weeks (DTT: 92.6% and JASP-EMT: 91.45%). Moreover, for response monitoring using the CGI, the PIs, coordinators, and interventionists scored 20% of all baseline and week 6 timepoint sessions. Any discrepancies were discussed and consensus reached across all sites. For CGI-I, site raters agreed on the early response/slow response status with the interventionist over 95% of the reviewed cases. For the few cases in which the sites differed, the consensus score trended to the more conservative score (ie, slower responder status).

### Primary Aim

#### Primary Outcome (SCU).

The average change in SCU (baseline to end-of-treatment) was 11.17 to 14.51 for JASP-EMT vs 11.17 to 16.59 for DTT. There was no difference in average change in SCU, which means there was insufficient evidence to reject the study’s Primary Aim hypothesis of no difference in average change in SCU. Starting with JASP-EMT led to 2.08 fewer utterances, on average, relative to starting with DTT (95% CI = −6.15 to 2.00; *p* = .32; effect size [ES] = −0.1) at 16 weeks. Results were similar for SCU at follow-up (effect size = −0.09).

#### Secondary Outcomes.

Results were similar for the secondary outcomes (Table S2, available online). There were no clinically significant differences in NDW (estimate = −1.84; 95% CI = −4.17 to 0.49; ES = −0.15), IJA (estimate = −0.38; 95% CI = −2.47 to 1.70; ES = −0.05), IBR (estimate = 1.50; 95% CI = −1.84 to 4.84; ES = 0.12), child-initiated JE (estimate = 0.06; 95% CI = −0.02 to 0.14; ES = 0.21), and play diversity (estimate = −0.62; 95% CI = −3.01 to 1.78; ES = −0.06) from baseline to treatment end (week 16).

### Secondary Aim

**FIGURE 2:**
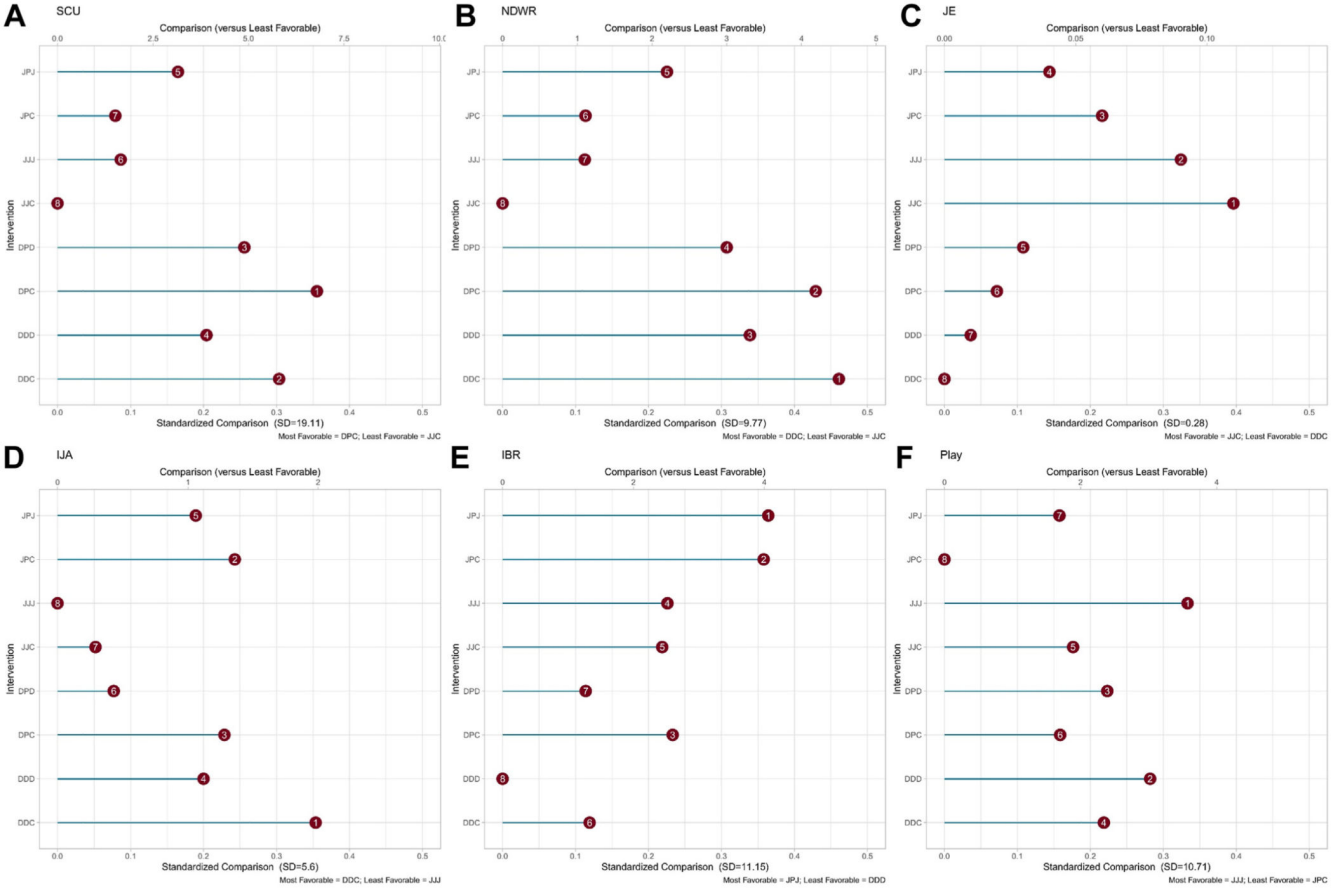
Secondary Aim Results Comparing the Individual Adaptive Intervention (AI) With the Least Favorable AI by Outcomes Note: The top scale shows the difference in outcome values between individual adaptive intervention (AI) with the least favorable adaptive intervention. The bottom scale shows the effect size comparing individual adaptive intervention with the least favoriable adaptive intervention (scaled by SD of outcome).

Figure 2 summarizes the results. A unique 3-letter initial (eg, JPC) is used to refer to each of the 8 (adaptive) interventions (Table 1): The first letter denotes stage 1 intervention; the second letter denotes stage 2 intervention for early responders; and the third letter denotes stage 2 intervention for slower responders. For example, JPC denotes the adaptive intervention that begins with JASP-EMT, provides parent training (P) to children responding early; and provides combined JASP-EMT+ DTT for children responding more slowly. Among parents who received parent training, 96% were mothers.

#### Primary Outcome (SCU).

Results do not support the prespecified hypothesis that JPC is the most favorable of the 8 interventions on SCU. JPC had an average SCU of 13.57 (95% CI = 8.72–18.43) at week 16, ranking it seventh (of 8). Instead, DPC (stage 1 DTT, stage 2 parent training for early responders, and stage 2 combined JASP-EMT+DTT for slower reponders) was estimated as most favorable intervention (estimate = 18.85; 95% CI = 12.90–24.80), and JJC the least favorable (estimate = 12.06; 95% CI = 8.17–15.96). The average difference between DPC and JJC was 6.78 SCU (95% CI = −0.343 to 13.92; ES = 0.37).

#### Secondary Outcomes.

For NDW, adaptive intervention DDC was the most favorable intervention (estimate = 12.01; 95% CI = 7.98–16.04), and JJC the least favorable (estimate, 7.51; 95% CI = 4.89–10.14) at week 16. The average difference between DDC and JJC was 4.496 words (95% CI = −0.31 to 9.31; ES = 0.38).

For JE, JJC was the most favorable (estimate = 36.38%; 95% CI = 27.49%−45.28%) and DDC the least favorable (estimate = 25%; 95% CI = 18%−33%) at week 16. The average difference between JJC and DDC was 11% (95% CI = 0%−23%; ES = 0.39).

For IJA, DDC was the most favorable (estimate = 8.49; 95% CI = 6.02–10.95) and JJJ the least favorable (estimate = 6.51; 95% CI = 4.79–8.23) at week 16. The average difference between DDC and JJJ was 1.535 (95% CI = −1.12 to 4.19; ES = 0.22).

For IBR, JPJ was the most favorable (estimate = 24.95; 95% CI = 21.31−28.59) and DDD the least favorable (estimate = 20.89; 95% CI = 17.21–24.56) at week 16. The average difference between JPJ and DDD was 4.06 (95% CI = 1.96–6.17; ES = 0.33).

For Play Diversity, JJJ was the most favorable (estimate = 23.61; 95% CI = 21.25–25.98) and JPC the least favorable (estimate = 20.04; 95% CI = 17.65–22.43) at week 16. The average difference between JJJ and JPC was 3.57 (95% CI = 0.2–6.94; ES = 10.94).

### Additional Analyses

#### Proximal Effect of Stage 1 Intervention on Response Status.

There was an 8% difference (95% CI = −6% to 22%) in the estimated effect of stage 1 JASP-EMT vs DTT on the probability of early response at week 6.

#### Interaction Effect Between the Intervention Decisions at Stages 1 And 2.

**FIGURE 3:**
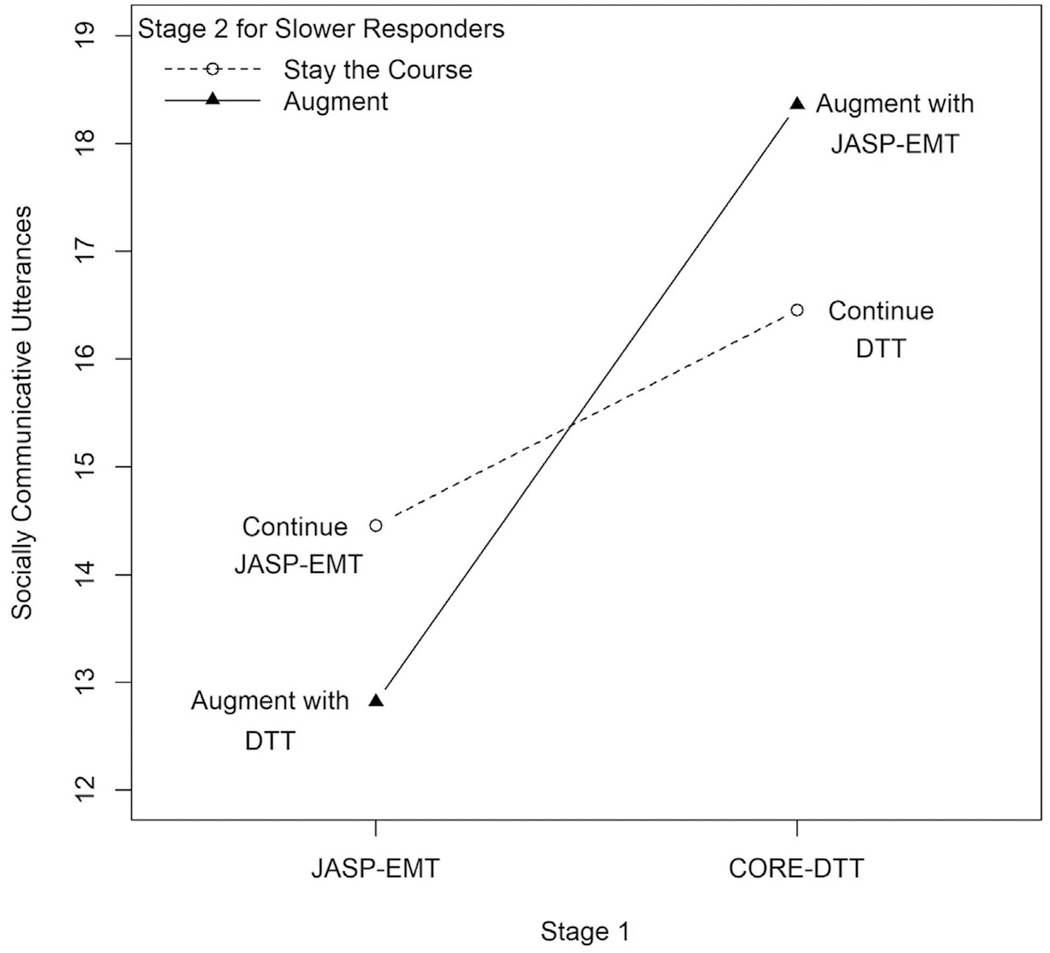
Comparison of Adaptive Interventions (AIs) That Augmented Stage 2 Intervention for Slower Responders With AIs That Stayed the Course for Spontaneous Communcative Utterances (SCU) at Week 16

The estimated 2-way interaction effect between stage 1 DTT and the stage 2 decision to augment for slower responders was 3.54 SCU (95% CI = 0.13–6.96) (Figure 3; Supplement 6, available online).

### Adverse Events

Seven severe adverse events occurred in 6 children but were not related to the study (ie, child injured himself/herself at home/school and needed hospital visits [n = 3], and hospitalization with stomach/bowel issues [n = 4]). Eleven nonsevere adverse events in 8 children were documented (ie, child had self-injurious behaviors or injured self during session [bit/bumped self or tripped over chair]).

## DISCUSSION

This sequentially randomized trial (AIM-ASD) was designed to answer several scientific questions related to the optimization of a 2-stage, 16-week adaptive intervention (involving JASP-EMT, DTT, early response monitoring, and Parent Training) for MV school-aged children. This article reports the results for the trial’s primary aim and a closely related secondary aim. The primary aim tested the effect of starting the intervention with JASP-EMT vs with DTT. The secondary aim was to estimate the most favorable of the 8 interventions (per outcome).

There are 3 main conclusions regarding these 2 aims. First, there is no evidence to reject the hypothesis of no difference between starting with JASP-EMT vs DTT on SCU. Results were similar at follow-up and for the secondary outcomes.

Second, of the 8 interventions compared, the most favorable in terms of average SCU at week 16 was DPC. This result is counter to the prespecified hypothesis (JPC most favorable). The 2 interventions (DPC and JPC) have more in common than not. Both offer parent training to children who are early responders, and both offer combined DTT+JASP-EMT to children who are slower responders. The 2 interventions differ only in terms of the provision of stage 1 intervention: DPC starts with DTT, whereas JPC starts with JASP-EMT.

The secondary aim results lend empirical support for the rationale that adaptive interventions may be ideally suited for capitalizing on the wide heterogeneity of autistic children. For all outcomes, with the exception of play (where JJJ was most favorable), the most favorable intervention was an adaptive intervention; and for 4 of the 6 outcomes considered, JJJ or DDD was among the least 2 favorable interventions.

Not surprisingly, the results of the secondary aim were similar for SCU and NDW (Figure 2a, b); these measures are known to be highly correlated, as NDW is a subset of the counts used to calculate SCU. Although there was no evidence to reject the primary aim hypothesis, for both of these outcomes, the 4 most favorable interventions started with DTT.

The 4 most favorable interventions for social communication outcomes, such as ioint engagment and requesting, all start with JASP-EMT. This finding is consistent with JE’s role as a primary mechanism for JASP-EMT’s relation to social communication and language outcomes.^6^ However, although increased JE has led to more IJA and language skills downstream in general samples of autistic children,^6^ it may be that MV children need greater intensity or more time to show benefits of increased engagement and IJA in this sample of MV children.

Third, results have implications for guiding intervention development for MV children with autism. Although a greater percentage of children who were offered stage 1 JASP-EMT were identified as early responders by the end of week 6, the most favorable strategy in the longer term (DPC) starts with DTT and augments with JASP-EMT for slower responders. Stage 1 DTT provided a boost of 3.54 SCU (1 word per 6 minutes) to the effect of augmenting (vs staying the course) for slower responders. This synergistic interaction effect is attributable to both the positive effect of augmenting with JASP-EMT for slower responders to stage 1 DTT and the negative (deleterious) effect of augmenting with DTT for slower responders to stage 1 JASP-EMT.

The findings that (1) DPC was most favorable and (2) there is a moderate synergistic effect of stage 1 DTT followed by stage 2 JASP-EMT for slower responders are especially interesting, given that stage 1 DTT had an 8% greater chance of identifying slower responders at week 6 relative to stage 1 JASP-EMT. We considered different explanations for this delayed but beneficial effect of DPC. One possibility is that structured teaching via DTT has a priming effect, providing the initial scaffolding needed, with JASP-EMT boosting responses if children are responding slowly. There is some precedent for this rationale based on earlier JASPER studies.^9,41^ A second possibility is that stage 1 DTT has a prescriptive effect, eliciting important information on which the therapist is able to capitalize (to the benefit of slower-responding children) during stage 2 JASP-EMT.

A study strength was providing intervention in the childrens’ natural environment, mostly at school, but also at home via the parent training component. Relative to clinic-based interventions, we expect this to have reduced burden on families, and this may be the reason that intervention attrition was so low. In addition, intervening in the school setting ensures that we can reach a more racially, ethnically, and income diverse sample of children, thus increasing representation across the autism spectrum. Limitations of the study include the short time frame (4 months), which may have been too brief to find change, and the difficulty in determining a meaningful change metric for this population. It is possible that setting our change metric at 13 SCU was too high for these children, who were more delayed in receptive language than in a previous study.^1^ Another limitation is that the 2 interventions, both focusing on similar goals, may have been more similar in execution than planned, as found in a study of toddlers.^42^ We note, too, the difficulty in current training approaches that rarely teach therapists a variety of intervention models; thus, it would be rare for a therapist currently to be able to integrate JASP-EMT and DTT in the community.^43^ Future training efforts might consider expanding the intervention tools available to community therapists.

### Implications for Clinical Practice

The secondary aim result that DPC led to the greatest gains in SCU provides preliminary guidance for how clinicians can use the 4 intervention components−DTT, JASP-EMT, parent training, and DTT+JASP-EMT−(ie, within a 2-stage, adaptive intervention) to maximize spoken communication in children with autism who are MV. For children 4.5 to 8 years of age (who score <1 year on a standardized expressive language scale), spoken language improved from 11.17 SCU at baseline to 18.85 SCU at week 16 (average change = 7.68; 95% CI = 2.13–13.24) with DPC.

However, this magnitude of change on DPC (average gain of 1 utterance per month) was modest. Furthermore, in comparison to the other 7 interventions, effect sizes were small to moderate. This result, and the finding that adaptive interventions generally had better outcomes compared to nonadaptive interventions, highlights the importance of this optimization trial and its planned future analyses.

### Planned Future Analyses

As this is an optimization trial (not an evaluation trial)^3,44^ planned, future analyses with this data set will investigate updates to each of the 3 intervention decision rules, with the aim of proposing an AI that leads to the greatest improvement in SCU.^45^ To inform the first decision, that is, between stage 1 DTT and JASP-EMT at baseline, the study will investigate whether baseline child characteristics of play and social communication skills moderate the effect of stage 1 intervention on children’s SCU. We hypothesized that children with more combination play and more joint attention gestures at baseline and receiving JASP-EMT would experience greater improvement in SCU compared to children who received DTT. For the second and third decision rules, which can use any data available up to the start of week 6 (including baseline data, the stage 1 decision rule, and change during stage 1), we will investigate whether parent expectations for intervention moderate the decision to offer parent training to early responders, and whether to offer combined DTT+JASP-EMT to slower responders.

## Supplementary Material

Supplemental Material 2

Supplemental Material

## Figures and Tables

**TABLE 1:** The Eight Interventions Embedded in the Sequential, Multiple-Assignment, Randomized Trial (SMART)

Intervention^a^		Adaptive intervention?	Stage 1	Tailoring variable at end of week 6	Stage 2	Cell
1	JJJ	No	JASP-EMT	Early responder	JASP-EMT	A+C
				Slower responder		
2	JJC	Yes	JASP-EMT	Early responder	JASP-EMT	A+D
				Slower responder	JASP-EMT + DTT	
3	JPJ	Yes	JASP-EMT	Early responder	JASP-EMT + Parent Training	B+C
				Slower responder	JASP-EMT	
4	JPC	Yes	JASP-EMT	Early responder	JASP-EMT + Parent Training	B+D
				Slower responder	JASP-EMT + DTT	
5	DDD	No	DTT	Early responder	DTT	E+G
				Slower responder		
6	DDC	Yes	DTT	Early responder	DTT	E+H
				Slower responder	DTT + JASP-EMT	
7	DPD	Yes	DTT	Early responder	DTT + Parent Training	F+G
				Slower responder	DTT	
8	DPC	Yes	DTT	Early responder	DTT + Parent Training	F+H
				Slower responder	DTT + JASP-EMT	

**Note**: DTT = Discrete Trials Training; JASP+EMT = Joint Attention, Symbolic Play, Engagement Regulation blended with Enhanced Milieu Teaching.

a The eight 2-stage interventions embedded in the trial, by design. All are adaptive interventions except for interventions 1 (JJJ) and 5 (DDD; offer Discrete Trials Training [DTT] only across both stages). JJJ is not an adaptive intervention because it offers Joint Attention, Symbolic Play, Engagement Regulation blended with Enhanced Milieu Teaching [JASP-EMT] across both stages regardless of response status at the end of week 6. DDD is not an adaptive intervention because it offers DTT across both stages regardless of response status at the end of week 6.

Three-letter abbreviation denoting each of the 8 interventions. The first letter denotes stage 1 intervention; there are 2 choices: J = JASP-EMT or D = DTT. The second letter denotes stage 2 intervention among early responders; there are 3 choices: J = continue JASP-EMT, P = add Parent Training, or D = continue DTT.

**TABLE 2:** Child Characteristics by Stage 1 (Initial) Intervention

Child characteristics	DTT (n = 98)	JASP-EMT (n = 96)
Biological sex, n (%)		
Female	20 (20.41)	20 (20.83)
Male	78 (79.59)	76 (79.17)
Age, mo	6.12 (1.23)	6.05 (1.30)
Ethnicity, n (%)		
Hispanic/Latino	29 (30)	28 (30)
Not Hispanic or Latino	68 (70)	66 (70)
Race, n (%)		
African American	7 (7.14)	11 (11.58)
Asian	10 (10.2)	5 (5.26)
Do not wish to respond	15 (15.31)	18 (18.94)
Other/multiracial	11 (11.22)	13 (13.68)
White	55 (56.12)	48 (50.53)
ADOS severity score	7.41 (1.21)	7.31 (1.43)
Leiter-R		
Brief IQ	61.06 (18.46)	59.47 (17.33)
Age equivalency	40.38 (14.37)	39.75 (13.60)
Mullen		
Visual reception	24.27 (8.93)	25.29 (9.91)
Fine motor	22.42 (10.84)	25.76 (13.05)
Receptive language	16.73 (9.06)	16.42 (8.67)
Expressive language	13.27 (8.87)	12.24 (8.3)
Mother’s education, n (%)		
Graduate/professional training	24 (24.49)	20 (21.05)
College graduate	39 (39.8)	31 (32.63)
Some college	16 (16.33)	18 (18.95)
Special training after high school	6 (6.12)	6 (6.32)
High school graduate or less	12 (12.24)	20 (21.05)
Do not wish to respond	1 (1.02)	0 (0)
Site, n (%)		
CornelleWeill Medical Center	25 (25.51)	23 (23.96)
UCLA	29 (29.59)	28 (29.17)
University of Rochester	20 (20.41)	21 (21.88)
Vanderbilt University	24 (24.49)	24 (25.00)

**Note**: ADOS = Autism Diagnostic Observation Schedule; DTT = Discrete Trials Training; JASP+EMT = Joint Attention, Symbolic Play, Engagement Regulation blended with Enhanced Milieu Teaching.

## Data Availability

Deidentified participant data will be available with publication in the The National Institute of Mental Health Data Archive (NDA). The data will be made available in the NDA for a specified purpose after approval of a proposal and signed data access agreement; however, data are available in the NDA. Wendy Shih and Daniel Almirall served as the statistical experts for this research. Tristram Smith, an original site PI, died prior to manuscript submission. The authors thank the families who participated in the studies and the many staff and graduate students who collected and coded data.
