# Supplementary material for: Adaptive Intervention for School-Age, Minimally Verbal Children With Autism Spectrum Disorder in the Community: Primary Aim Results
Source: J Am Acad Child Adolesc Psychiatry. Author manuscript; Available in PMC 2025 Nov 5. (PMC12587618; doi:10.1016/j.jaac.2024.10.020)
Supplement: Supplemental Material 2 [file NIHMS2110500-supplement-Supplemental_Material_2.docx]

**Table S1: Fidelity to Intervention Components Across all Stages**

| **Intervention Component** | **Fidelity (% of 100)** |
| --- | --- |
| DTT: Total | 92.60 |
| Student Readiness | 93.87 |
| Verbal Instruction | 97.20 |
| Reinforcement/Correction Procedure | 86.49 |
| JASP-EMT: Total | 91.45 |
| Basic Strategies | 95.38 |
| Play Routines | 90.27 |
| Joint Attention/Requesting Skills | 80.00 |
| SGD-EMT | 97.57 |

**Table S2** Primary aim results on the effect of starting with JASP-EMT vs starting with DTT

|  |  | **JASP-EMT** | | | **DTT** | | | **Treatment Effect at Week 16** | | **Treatment Effect at Week 32** | |
| --- | --- | --- | --- | --- | --- | --- | --- | --- | --- | --- | --- |
| **Outcome** | **Baseline** | **Week 6** | **Week 16** | **Week 32** | **Week 6** | **Week 16** | **Week 32** | **Estimate** | **ES*** | **Estimate** | **ES*** |
| **Primary Outcome** |  |  |  |  |  |  |  |  |  |  |  |
| **SCU** | 11.17 | 14.76 | 14.51 | 14.63 | 13.35 | 16.59 | 16.31 | -2.07 | -0.1 | -1.68 | -0.09 |
| **Secondary Outcomes** |  |  |  |  |  |  |  |  |  |  |  |
| **NDW** | 6.43 | 8.09 | 9.03 | 9.57 | 8.66 | 10.87 | 10.39 | -1.83 | -0.15 | -0.82 | -0.07 |
| **IJA** | 6.81 | 8.19 | 7.25 | 7.76 | 7.57 | 7.63 | 7.51 | -0.38 | -0.05 | 0.25 | 0.004 |
| **IBR** | 19.46 | 21.43 | 23.91 | 21.71 | 21.63 | 22.41 | 22.01 | 1.5 | 0.12 | -0.31 | -0.02 |
| **JE** | 0.3 | 0.25 | 0.33 | 0.3 | 0.27 | 0.27 | 0.28 | 0.06 | 0.21 | 0.02 | 0.09 |
| **Play Diversity** | 20.12 | 22.37 | 21.84 | 20.631 | 21.15 | 22.45 | 21.99 | -0.62 | -0.06 | -1.68 | -0.15 |

^* Effect size is defined as the average difference between JASP-EMT and DTT at week 16 divided by the pooled standard deviation at week 16.^
