# Supplementary material for: Adaptive Intervention for School-Age, Minimally Verbal Children With Autism Spectrum Disorder in the Community: Primary Aim Results
Source: J Am Acad Child Adolesc Psychiatry. Author manuscript; Available in PMC 2025 Nov 5. (PMC12587618; doi:10.1016/j.jaac.2024.10.020)
Supplement: Supplemental Material [file NIHMS2110500-supplement-Supplemental_Material.docx]

**Supplement 1**

**Intervention Components**

Each of DTT, JASP-EMT and DTT+JASP-EMT was delivered at the child’s school, 4 days per week, for approximately forty-five minutes daily. Parent training was conducted in the family home by the interventionist assigned to the child in stage 2.

*Discrete Trial Training (DTT).* DTT is based on behavioral learning theory in which communication and related skills are taught through systematic direct instruction^1^. The goal of DTT is to help children be successful in learning communication skills by breaking these skills down into small steps, providing systematic direct instruction on each step, and reinforcing children (e.g., with praise or access to preferred items) for demonstrating skills. Imitation and attention skills are a focus early in intervention. DTT is the most common evidence-based approach for teaching children with autism and is often considered the closest to a “standard of practice” for the field. The participants in the proposed study had at least 1 year of previous intervention most often in an ABA (applied behavior analytic) program with DTT as a main strategy. While many children were exposed to DTT prior to entering this trial, it was believed important to ensure that they (a) received quality DTT, and (b) had exposure to CORE elements related to language learning, specifically social communication and play targets in this study, to make the comparison with JASP-EMT. Tablet based pictures were also used to support language goals, similar to the commonly used picture exchange systems in community practice.

*Joint Attention, Symbolic Play, Engagement & Regulation blended with Enhanced Milieu Teaching (JASP+EMT)*. JASP-EMT is a developmentally anchored behavioral intervention that assumes that communication develops from social interactions in which specific social engagement strategies, symbolic representations, and early communication forms are modeled and naturally reinforced by adult partner responses to the child^2^. The goal of JASP-EMT is to increase (a) joint engagement, (b) joint attention and requesting gestures, (c) functional and symbolic play involving objects and persons, and (d) verbal and nonverbal communication by facilitating meaningful social interactions. The social interaction foundation of JASP-EMT is crucial where modeling and expansions of communicative behaviors are used strategically within meaningful social interactions with interventionists and caregivers. For minimally verbal children with autism, meaningful social interaction is needed for establishing the platform on which language input and developmental skills will be built. Unlike DTT, JASP-EMT was a novel intervention to which few children had previous exposure. Tablet based language support (proloquo2go software) was integrated into intervention sessions similar to previously published JASP-EMT research^8^.

*DTT + JASP-EMT*. This intervention includes strategies used in each of DTT and JASP-EMT. In each 45-minute session, using a dashboard of potential intervention strategies, DTT and JASP-EMT were both applied to varying amounts depending on the need of child^4^. Thus, in some cases DTT was used to prime the intervention targets by teaching the child directly using a structured didactic approach, and then JASP-EMT was implemented to explicitly promote generalization of the target skills. This hybrid approach (prime then social practice) has some precedent as used in an earlier trial with preschool-aged children with autism^3^. Other children may have received more of one approach versus another depending on their first line intervention, and the interventionist’s clinical judgment based on the dashboard. This multi-component intervention requires a interventionist highly-trained in both DTT and JASP-EMT (and in how to tradeoff session to session decisions on employing the two approaches) and thus, DTT + JASP-EMT represents a significantly greater intervention cost, thereby justifying it as a stage 2 intervention to be used only with slower responders.

*Parent training*. Parent training is conducted in the family home by the child’s primary interventionist using a combination of discussion, modeling, and live coaching support for the stage 1 intervention model (DTT or JASP-EMT). With parent training, the goal was to capitalize on early gains with school intervention to generalize intervention effects to the home, i.e., beyond the school-therapist context. In parent training with JASP-EMT, parents learned social engagement and language strategies that support children’s joint attention, play, and language. In parent training with DTT, parents learned instructional strategies to support teaching specific target communication behaviors. Both JASP-EMT and DTT have been previously implemented with a parent training component^5,6^. In practice, providing parent training in the home or school as requested by parents (in addition to providing DTT or JASP-EMT at the school) represents a significant cost/burden, both to parents and to providers, thereby justifying it as a stage 2 intervention to be used only with early responders.

*Intervention participation/engagement plan*. Parents (or primary caregivers) are provided progress notes from their primary interventionist and based on the communication log kept by each interventionist at each site. This intervention component is embedded as part of DTT, JASP-EMT, and DTT + JASP-EMT, but not Parent Training. This was done to inform and engage parents since the interventions (except for Parent Training) were carried out primarily in schools and not homes.

*Early response (slow response) monitoring*. Early response versus slower response categorization at week 6 was based on a 7-item Clinical Global Impressions-Improvement scale (CGI-I^7^). The CGI-I was rated by the interventionist at week 6, and based on change in CGI-severity rating at baseline and week 6, informed by the interventionist’s weekly data and clinical judgement. “Early response” was defined as CGI-I values of 1 or 2 at week 6; “slower response” was defined as CGI-I values of 3 to 7 at week 6. If a child was not available to provide the week 6 CGI measure, the child’s previous known session was used to make the week 6 classification. The CGI-I is a relatively easy-to-use measure of clinical progress that is common in other areas of psychiatry^8^. The interventionist providing the stage 1 intervention (JASP-EMT or DTT) provided the CGI-I rating.

**Supplement 2**

**Fidelity Monitoring**

Prior to the beginning of the study, interventionists at all 4 sites were trained to high levels of fidelity (>80% on all intervention elements and quality indicators, based on a rating scale) with 2-3 children (these children were not part of the randomized study but were minimally verbal). Each site had a research coordinator. Interventionists at each site were supervised by the site coordinator via weekly individual or group meetings. For each interventionist, the coordinator used a fidelity rating form to rate a random sample of 20% of video recorded sessions. The feedback was shared with the interventionist via email and all participants were discussed during weekly cross-site conference calls that focused on updates, exploring new programs/routines, troubleshooting and insure consistency of intervention across interventionists and sites. If levels of fidelity <80% were observed (for any one coordinator rating), the interventionist received weekly review of video recorded sessions and additional training if necessary, until fidelity returned to 80% or greater.

Prior to the beginning of the study (or the start of an interventionist’s first participating child in the case of those joining throughout the study), interventionists were trained on the CGI-I used to determine early/slow response at week 6. During the trial, all week-6 CGI-I measures were scored by the interventionist, and consensus coded by the PI and/or site coordinator overseeing the interventionist at each site. In addition, 20% of week-6 CGI-I measures (across all sites and children) were selected and scored by PIs and coordinators across all sites. In cases of disagreement, the final decision was based on consensus in a group discussion.

**Supplement 3**

**Research Measures**

*Eligibility and stratification measures*. Inclusion/exclusion criteria and measures used for the initial stratified randomization were based on: clinical diagnoses of autism validated by research reliable administration of the (ADOS-2^9^); the Leiter-R^10^ or MSEL^11^ if the basal could not be obtained on the Leiter-R , a natural language sample (NLS) for the SCU and NDW; and demographic, medical, genetic, physical and intervention history questionnaires completed by parents at baseline.

*Natural language sample (NLS^5^).* The NLS was collected at baseline, week 6, exit, and follow up. An independent evaluator (IE), who was blind to intervention assignment, conducted the NLS; further, the child’s interventionist was not present during the NLS. The NLS is a 21-minute standardized, video recorded, naturalistic adult-child interaction The NLS was later transcribed using SALT conventions (Systems Analysis of Language Transcripts^1,2^) and each verbal and nonverbal behavior was coded by an IE using standard coding protocol to derive the primary outcome variable (see below) and several other language variables.

For the language sample assessments, we have conducted a test/retest reliability with this sample in which the assessment was conducted twice within a span of 1 week. For both SCU and NDWR, the correlation between the test/retest are highly and significantly correlated (0.72 and 0.81 respectively).The average intra-class correlation (ICC) for transcript coding was among five coders (NDWR ICC = 0.97; SCU ICC = 0.99).

Primary research outcome. The primary outcome is an IE-collected and -coded measure of total spontaneous socially communicative utterances (SCU) based on the NLS, a continuous measure. SCUs are unprompted, generated (non-scripted) verbal utterances that are directed to another person for the purpose of sharing information (comment) or making a request. This measure has been found to have high-reliability and sensitivity to intervention in previous studies^5^. SCU was collected at week 0 (baseline), week 6 (early response), week 16 (exit from intervention) and week 32 (follow up). The endpoint for the primary aim is at week 16 (end of treatment).

*Secondary research outcomes*. Five secondary outcomes were pre-specified: (a) child-initiated time jointly engaged (JE) with a caregiver, (b) total number of different word roots (NDW), (c) number of different play types across play levels-play diversity, (d) initiating joint attention (IJA), and (e) initiating behavioral request (IBR). First is the total time in a child initiated JE state^13^. JE is a duration measure taken from the video-recorded Caregiver-Child Interaction (CCX). The CCX was derived from the Communication Play Protocol (CPP^14^) including three 5-minute scenes adapted from the CPP including (a) free play, (b) hidden objects, and (c) drawing. The caregiver was read a brief script that asked them to make the child the star of the scene using the materials provided. After a scene was completed, the caregiver was provided with the next set of materials and read the script for that scene. The CCX was collected at weeks 0,6,16, and 32. Child initiated JE was coded at weeks 0, 6, 16, and 32 from the video recorded free play from the CCX at these time points.

Second, is the total number of novel distinct word roots (NDW), coded with SALT conventions from the transcribed NLS. NDW was coded at weeks 0,6,16 and 32.

Third, play diversity and play mastery level were coded from the video-recorded Structured Play Assessment-Revised (SPA-R^15,16^). Play diversity refers to the number of different types (unique appropriate actions on objects) within each play level and play level mastery refers to the highest level of play the child shows that meets mastery criteria ---defined as 3 different types within each level of play the child completes spontaneously during the SPA-R). The SPA-R is collected at weeks 0, 6, 16 and 32. Inter-rater reliability ranged from ICC = 0.61 to ICC = 0.94 (average 0.89).

Finally, IJA and IBR were collected from the ESCS (Early Social Communication Scales17-20). The ESCS is a 20-minute semi-structured assessment, collected at weeks 0, 6, 16, and 32 and was used to assess children’s joint attention (IJA) and behavior regulation skills (IBR). Inter-rater reliability was high: IJA (ICC = 0.82), IBR (ICC = 0.94).

All secondary outcome measures were coded from independently administered assessments and coded by researchers blind to intervention condition and time point.

*Study Sample Retention Plan*. All children, once randomized to stage 1 intervention were included in all analyses. Participants are eligible for compensation at each of the three major time points: baseline, exit, and follow-up. Participants are encouraged to complete all assessments at each time point. Even if participants do not complete all assessments at each time point, they are still eligible to receive their $25 compensation as long as assessments were attempted. Engagement efforts included text messages or phone calls to parents, paper reminders sent home in the child’s backpack, and notes to parents via the classroom teacher. These retention strategies were implemented by the site research team.

**Supplement 4**

**Pre-planned primary and secondary aim analyses and results**

All analyses for the study were pre-specified prior to data collection. Each section of this supplement leads with a description of the “Design” for each part of the primary and secondary aim analyses, written from the perspective of the trialist *prior* to data collection. Each section then includes an indented “Results” section, which details the results of that design, or points the reader to the appropriate section in the main narrative of the manuscript where the results are presented.

**Intent to Treat Sample**

*Design*: All subjects, once randomized, will be included in the intent-to-treat sample used in the Primary and Secondary Aim analyses.

*Results:* All children who were randomized were included in the intent-to-treat sample for data analysis.

**Missing Data**

*Design related to investigating missing data*: As in any study, missing values may occur in outcomes due to dropout or inability to reach a participant for follow-up. A thorough investigation of mechanisms for missing data will be carried out.

*Results related to investigating missing data*: No children had missing data for the primary outcome (language sample) at baseline. For SCU, less than 2% of outcomes were missing by study exit. For secondary language outcomes, less than 5% of the outcomes were missing by study exit. Missing data patterns were near identical across all outcomes (SCU, NDWR, IJA, IBR, engagement, play diversity). Thus, across all outcomes, children were categorized into two groups: missing any of the outcomes vs not missing any of the outcomes. Multiple logistic regression was then used to understand the relationship between the baseline covariates and outcomes with missing vs not missing Baseline covariates include site, race, ethnicity, gender and treatment group assignment, repetitive behaviors, object engagement, and non-verbal IQ. Site and race were found to be associated with “missingness”; however, site and race are heavily correlated. Hence, only site was later included in all analyses.

| **Logistic Models** | **Baseline Covariates** | **Odds Ratio** | **p-value** |
| --- | --- | --- | --- |
| 1 | Site 2 vs Site 1 | 1.441 | 0.394 |
| 1 | Site 3 vs Site 1 | 1.021 | 0.963 |
| 1 | Site 4 vs Site 1 | 0.259 | 0.011 |
| 2 | JASP-EMT vs DTT | 1.648 | 0.126 |
| 3 | Male vs Female | 0.989 | 0.977 |
| 4 | Hispanic vs Not Hispanic | 1.148 | 0.698 |
| 5 | Black vs Asian | 0.438 | 0.251 |
| 5 | Do not wish to disclose vs Asian | 0.315 | 0.074 |
| 5 | Other/Multi vs Asian | 0.230 | 0.042 |
| 5 | White vs Asian | 0.280 | 0.025 |
| 6 | Repetitive Behavior | 1.012 | 0.173 |
| 7 | Object Engagement | 0.594 | 0.482 |
| 8 | Non-verbal IQ | 1.013 | 0.262 |
| 9 | SCU | 1.000 | 0.999 |
| 10 | NDWR | 1.017 | 0.373 |
| 11 | Joint Attention Skills | 1.012 | 0.574 |
| 12 | Behavior Requesting skills | 1.013 | 0.383 |
| 13 | Child Initiated Joint Engagement | 0.655 | 0.570 |
| 14 | Play Diversity | 1.023 | 0.101 |

*Design related to the multiple imputations*: Missing data will be dealt with explicitly using multiple imputation (MI) procedures^21,22^. MI is a simulation-based approach to deal with incomplete data using plausible values to replace missing data. A sequential regression multivariate imputation algorithm was used to generate M=25 “complete data sets” where each data set contained different estimates of the missing values (no missingness). Covariates found to be related to missing will be used in the regression models. To implement multiple imputation, we will use the MICE (Multiple Imputation by Chain Equations) package^24^ for R CRAN Version 4.0.0 (R Core Team, 2014) to generate 25 imputed data sets.

*Results related to the multiple imputations*: Multiple imputations were conducted as planned.

**Data Analyses**

*Design of the primary aim analysis*: The Primary Aim analysis contrasted adaptive interventions beginning with JASP-EMT vs. interventions beginning with DTT (i.e., to evaluate the main effect of initial treatment) on change (increase) in SCU (the primary outcome) during the treatment phase. Similar models were fit for secondary outcomes. This is a comparison of cells A+B+C vs D+E+F (see Figure 1 and Table 1). Linear mixed models^25^ (LMM), also known as random effects or growth curve models), fitted with SAS PROC MIXED was used to analyze the longitudinal data. LMMs uses all available measurements, allowing subjects to have an unequal number of observations and producing unbiased parameter estimates as long as unobserved values are missing at random. The analysis fitted a LMM with fixed effects for the intercept, time, and a group by-time interaction term, where group is an indicator of phase-one treatment (JASP-EMT[1] vs. CORE-DTT[-1]; effect coding), for each of the 25 imputed sets. The group variable was not in the model because the baseline values for both JASP-EMT and DTT were expected to be equal as the study is a RCT. The LMM also included random effects for the intercept and an unstructured within-person correlation structure for the residual errors and will adjust for the following measures collected at baseline (pre-randomization): site, age, and non-verbal IQ. The primary contrast in this study is the between groups difference during treatment phase while controlling for site, child’s age at baseline, and non-verbal IQ. The LMM outputs from each of the 25 MI sets were later combined into one final inference using Barnard and Rubin (1999)^26^ recommended adjusted degrees of freedom. Estimates at each timepoint are available upon request.

Design of the secondary aim analysis: The Secondary Aim analysis estimated which of the 8 AI was most favorable at exit. The data analysis method of Robins and colleagues^27,28^ was used to contrast the adaptive interventions based on number of spontaneous socially communicative utterances to determine whether any of the interventions appear to be clearly better or worse than others. As part of the design, a child will contribute differentially to one or more of the 8 strategies (depending on the treatments to which he or she is randomized and whether he or she is an early or slow responder), requiring a weighted comparison. Specifically this analysis involves a weighted comparison of the cells A+C vs A+D vs B+C vs vs B+D vs E+G vs E+H vs F+G vs F+H; the method by Robins and colleagues (2000)^29^ weights each child using the known randomization probabilities. GEE models with the known randomization probabilities were utilized to estimate which of the 8 strategies was the best (i.e. most favorable) for each primary and secondary outcome. Estimates from the regression models for each of the 8 AI at exit (i.e. identify most and least favorable AI) are available upon reasonable request.

**Supplement 5**

**Interventionist Fidelity**

Supplement T1 shows high fidelity scores (percentage correct of 100), on average across all components, over the course of 16 weeks (DTT: 92.6 and JASP-EMT: 91.45). For DTT, Reinforcement/Correction Procedure component had the lowest fidelity score of 86.49 and the verbal instruction domain had the highest fidelity score of 97.20. For JASP-EMT, the joint attention and requesting skills domain had the lowest fidelity score of 80 and SGD-EMT/Language component had the highest score of 97.57.

**Supplement 6**

**Additional analyses**

*Proximal effect of stage 1 intervention on early response status*. There was an 8% difference (95%CI, -6% to 22%) in the estimated effect of stage 1 JASP-EMT vs DTT on the probability of early response at week 6. That is, as part of stage 1 DTT, 46% (45/98; 95%CI, 36% to 56%) of children are categorized as early responders; whereas, with stage 1 JASP-EMT, the estimate is 54% (52/96; 95%CI, 44% to 64%).

*Interaction effect between the intervention decisions at stage 1 and 2.* The estimated, two-way interaction effect between stage 1 DTT and the stage 2 decision to augment for slower responders was 3.54 SCU’s (95%CI, 0.13 to 6.96; Figure 3). That is, for adaptive interventions that start with DTT, the decision to augment for slower responders (with JASP-EMT) vs stay the course leads to 1.9 more SCUs (95%CI, -2.57 to 6.37). Whereas, for adaptive interventions that start with JASP-EMT, the decision to augment (with DTT) vs stay the course leads to 1.6 fewer SCUs (95%CI, -4.62 to 1.34).

**References**

1. Smith, T. (2001). Discrete trial training in the treatment of autism. Focus on autism and other developmental disabilities, 16(2), 86-92. [https://doi.org/10.1177/108835760101600](https://doi.org/10.1177/108835760101600204)
2. Kasari, C., Lawton, K., Shih, W., Barker, T. V., Landa, R., Lord, C., ... & Senturk, D. (2014). Caregiver-mediated intervention for low-resourced preschoolers with autism: An RCT. Pediatrics, 134(1), e72-e79. <https://doi.org/10.1542/peds.2013-3229>
3. Kasari, C., Freeman, S., & Paparella, T. (2006). Joint attention and symbolic play in young children with autism: A randomized controlled intervention study. Journal of child psychology and psychiatry, 47(6), 611-620.  <https://doi.org/10.1111/j.1469-7610.2005.01567.x>
4. Chorpita, B. F., Bernstein, A., Daleiden, E. L., & Research Network on Youth Mental Health. (2008). Driving with roadmaps and dashboards: Using information resources to structure the decision models in service organizations. Administration and Policy in Mental Health and Mental Health Services Research, 35(1-2), 114-123. https://doi.org/10.1007/s10488-007-0151-x
5. Kasari, C., Kaiser, A., Goods, K., Nietfeld, J., Mathy, P., Landa, R., ... & Almirall, D. (2014). Communication interventions for minimally verbal children with autism: A sequential multiple assignment randomized trial. Journal of the American Academy of Child & Adolescent Psychiatry, 53(6), 635-646. <https://doi.org/10.1016/j.jaac.2014.01.019>
6. Kasari, C., Shire, S., Shih, W., Landa, R., Levato, L., & Smith, T. (2023). Spoken language outcomes in limited language preschoolers with autism and global developmental delay: RCT of early intervention approaches. Autism Research.  <https://doi.org/10.1002/aur.2932>
7. Guy, W. (1976). Clinical global impressions scale. Psychiatry. [https://doi.org/10.1037/t48216-000](https://psycnet.apa.org/doi/10.1037/t48216-000)
8. Arnold, L. E., Vitiello, B., McDougle, C., Scahill, L., Shah, B., Gonzalez, N. M., ... & Tierney, E. (2003). Parent-defined target symptoms respond to risperidone in RUPP autism study: customer approach to clinical trials. Journal of the American Academy of Child & Adolescent Psychiatry, 42(12), 1443-1450. <https://doi.org/10.1097/00004583-200312000-00011>
9. Lord CLR, Gotham K, Guthrie W. Autism diagnostic observation schedule, second edition (ADOS-2) manual. Torrance, CA: Western Psychological Services; 2012.
10. Roid, G. H., & Miller, L. J. (1997). Leiter international performance scale-revised (Leiter-R). Wood Dale, IL: Stoelting, 10.
11. Mullen, E. M. (1995). Mullen scales of early learning (pp. 58-64). Circle Pines, MN: AGS.
12. Miller, J., & Chapman, R. (2012). Systematic analysis of language transcripts (SALT)[Computer software]. Middleton, WI: SALT Software LLC.
13. Adamson, L. B., & Bakeman, R. (2006). Development of displaced speech in early mother–child conversations. Child Development, 77(1), 186-200. <https://doi.org/10.1111/j.1467-8624.2006.00864.x>
14. Adamson, L. B., & Bakeman, R. (2016). The communication play protocol: capturing variations in language development. Perspectives of the ASHA special interest groups, 1(12), 164-171. <https://doi.org/10.1044/persp1.SIG12.164>
15. Kasari, C., Freeman, S., & Paparella, T. (2006). Joint attention and symbolic play in young children with autism: A randomized controlled intervention study. Journal of child psychology and psychiatry, 47(6), 611-620. <https://doi.org/10.1111/j.1469-7610.2005.01567.x>
16. Sigman, M., & Ungerer, J. A. (1984). Cognitive and language skills in autistic, mentally retarded, and normal children. Developmental psychology, 20(2), 293. [https://doi.org/10.1037/0012-1649.20.2.293](https://psycnet.apa.org/doi/10.1037/0012-1649.20.2.293)
17. Mundy, P., Sigman, M., Ungerer, J., & Sherman, T. (1986). Defining the social deficits of autism: The contribution of non‐verbal communication measures. Journal of child psychology and psychiatry, 27(5), 657-669.  <https://doi.org/10.1111/j.1469-7610.1986.tb00190.x>
18. Mundy, P., Sigman, M., & Kasari, C. (1994). Joint attention, developmental level, and symptom presentation in autism. Development and Psychopathology, 6(3), 389-401. <https://doi.org/10.1017/S0954579400006003>
19. Mundy, P. (1995). Joint attention and social-emotional approach behavior in children with autism. Development and Psychopathology, 7(1), 63-82. <https://doi.org/10.1017/S0954579400006349>
20. Seibert, J. M., Hogan, A. E., & Mundy, P. C. (1982). Assessing interactional competencies: The early social‐communication scales. Infant mental health Journal, 3(4), 244-258. [https://doi.org/10.1002/1097-0355(198224)3:4<244::AID-IMHJ2280030406>3.0.CO;2-R](https://doi.org/10.1002/1097-0355(198224)3:4%3C244::AID-IMHJ2280030406%3E3.0.CO;2-R)
21. Little, R. J., & Rubin, D. B. (2019). Statistical analysis with missing data (Vol. 793). John Wiley & Sons.
22. White, I. R., Royston, P., & Wood, A. M. (2011). Multiple imputation using chained equations: issues and guidance for practice. Statistics in medicine, 30(4), 377-399. <https://doi.org/10.1002/sim.4067>
23. Harel, O. (2009). The estimation of R 2 and adjusted R 2 in incomplete data sets using multiple imputation. Journal of Applied Statistics, 36(10), 1109-1118. <https://doi.org/10.1080/02664760802553000>
24. Van Buuren, S., & Groothuis-Oudshoorn, K. (2011). mice: Multivariate imputation by chained equations in R. Journal of statistical software, 45, 1-67. [10.18637/jss.v045.i03](https://doi.org/10.18637/jss.v045.i03)
25. Verbeke, G., Molenberghs, G., & Verbeke, G. (1997). Linear mixed models for longitudinal data (pp. 63-153). Springer New York.
26. Barnard, J., & Rubin, D. B. (1999). Miscellanea. Small-sample degrees of freedom with multiple imputation. Biometrika, 86(4), 948-955. <https://doi.org/10.1093/biomet/86.4.948>
27. Orellana, L., Rotnitzky, A., & Robins, J. M. (2010). Dynamic regime marginal structural mean models for estimation of optimal dynamic treatment regimes, part I: main content. The international journal of biostatistics, 6(2). <https://doi.org/10.2202/1557-4679.1200>
28. Robins, J.M. (2008). Causal models for estimating the effects of weight gain on mortality. International Journal of Obesity, 32(S15-S41) https://doi.org/10.1038/ijo.2008.83
29. Robins, J. M., Hernan, M. A., & Brumback, B. (2000). Marginal structural models and causal inference in epidemiology. Epidemiology, 550-560.
